# *Ntrk1* mutation co-segregating with bipolar disorder and inherited kidney disease in a multiplex family causes defects in neuronal growth and depression-like behavior in mice

**DOI:** 10.1038/s41398-020-01087-8

**Published:** 2020-11-24

**Authors:** Kazuo Nakajima, Alannah Miranda, David W. Craig, Tatyana Shekhtman, Stanislav Kmoch, Anthony Bleyer, Szabolcs Szelinger, Tadafumi Kato, John R. Kelsoe

**Affiliations:** 1 Laboratory for Molecular Dynamics of Mental Disorders, RIKEN Center for Brain Science, Saitama, Japan; 2 Department of Psychiatry, University of California San Diego, San Diego, USA; 3 Department of Genetics, University of Southern California, Los Angeles, USA; 4 Research Unit for Rare Diseases, Department of Pediatrics and Adolescent Medicine, First Faculty of Medicine, Charles University, Prague, Czechia; 5 Section on Nephrology, Department of Internal Medicine, Wake Forest School of Medicine, Winston-Salem, NC 27157 USA; 6 Neurogenomics Division, Translational Genomics Research Institute, Arizona, USA; 7 Department of Psychiatry, Juntendo University, Tokyo, Japan; 8 Institute for Genomic Medicine, University of California San Diego, San Diego, USA

**Keywords:** Stem cells, Molecular neuroscience, Bipolar disorder

## Abstract

Previously, we reported a family in which bipolar disorder (BD) co-segregates with a Mendelian kidney disorder linked to 1q22. The causative renal gene was later identified as *MUC1*. Genome-wide linkage analysis of BD in the family yielded a peak at 1q22 that encompassed the *NTRK1* and *MUC1* genes. *NTRK1* codes for TrkA (Tropomyosin-related kinase A) which is essential for development of the cholinergic nervous system. Whole genome sequencing of the proband identified a damaging missense mutation, E492K, in *NTRK1*. Induced pluripotent stem cells were generated from family members, and then differentiated to neural stem cells (NSCs). E492K NSCs had reduced neurite outgrowth. A conditional knock-in mouse line, harboring the point mutation in the brain, showed depression-like behavior in the tail suspension test following challenge by physostigmine, a cholinesterase inhibitor. These results are consistent with the cholinergic hypothesis of depression. They imply that the NTRK1 E492K mutation, impairs cholinergic neurotransmission, and may convey susceptibility to bipolar disorder.

## Introduction

Bipolar disorder (BD) is a major psychiatric disorder that occurs in around 1% of the population and is associated with a 5–17% rate of suicide^1^. The heritability of BD is 0.70, and genome-wide association studies (GWAS), conducted by a large international consortium, have identified over 50 significant loci^2,3^. However, single nucleotide polymorphisms (SNP) identified by GWAS are common variants with very small effect sizes^2^. Recently, whole genome and exome sequencing have made it possible to examine rare variants with larger effects that might explain some of the “missing heritability”^4,5^. A possible advantage of rare variants is that the larger effect size provides stronger evidence to justify conducting studies of the variant’s effect on function using animal and cellular models. In this regard, unusual rare families in which BD co-segregates with a Mendelian illness can be useful in mapping and in identifying mutations of strong effect. In such cases, the causative gene for the Mendelian disease may cause bipolar disorder as a pleiotropic effect or be linked with another gene for bipolar disorder. Bipolar disorder is reported to co-segregate with several Mendelian diseases^6–8^.

We previously identified a large pedigree, in which bipolar disorder co-segregated with autosomal dominant tubulo-interstitial kidney disease (ADTKD)^9^. ADTKD is a rare autosomal dominant disorder characterized by tubulointerstitial fibrosis, with chronic kidney disease progressing variably to end-stage kidney disease between 20 and 80 years. The responsible gene for ADTKD in this family, was identified as *MUC1* at 1q22 encoding *Mucin 1*, a protein that forms protective mucous barriers on the surfaces of epithelial cells and is involved in cell signaling^10^. The function of MUC1 and its lack of expression in the brain suggest that the gene for BD is likely linked to MUC1.

We, therefore performed linkage and whole genome sequencing analysis of this family and identified a E492K mutation in the *NTRK1* gene that is approximately 1 Mb distal to MUC1. Tropomyosin receptor kinase A (TrkA), the protein encoded by *NTRK1*, is a receptor for nerve growth factor (NGF), which is important for development and function of cholinergic neurons in autonomic nerves and the brain.

Several decades of work implicate the role of acetylcholine (ACh) in depression. High rates of depression in crop dusters exposed to cholinesterase inhibitors (resulting in increased ACh) was one of the first clues. Subsequently, it was shown that infusion of physostigmine, another cholinesterase inhibitor into recently remitted patients with both unipolar and bipolar depression resulted in a brief return of their depression^11^. REM sleep is regulated by a norepinephrine (NE)–ACh feedback loop in the pons. One of the more robust endophenotypes for depression is a shortening of the time to onset of REM or dreaming sleep. By infusing a muscarinic agonist into sleeping subjects in the sleep lab, the time to REM onset was shortened. Depressed or recently remitted patients demonstrated a super-sensitivity to this effect and a greater shortening of REM than controls^12^. More recently, N-methyl scopolamine (NMS), a muscarinic antagonist has been shown to have rapid antidepressant effects similar to ketamine^13^. Together these data argue that a factor in susceptibility to bipolar disorder and depression is a hypersensitivity to ACh via muscarinic receptors.

In light of these data supporting cholinergic hypersensitivity, the identification of a likely functional variant in the *NTRK1* gene suggested the hypothesis that defects in TrkA lead to a defective cholinergic system, which in turn predisposes to mood disorders. Towards testing this hypothesis, we verified the functional alterations caused by this mutation using cellular models. Finally, we generated conditional knock-in mice carrying this mutation specifically in the brain and showed depression-like behavior in response to physostigmine, which activates cholinergic signaling. These findings altogether suggest that *NTRK1* mutation linked to ADTKD and bipolar disorder contribute to the bipolar phenotype in this family.

## Methods

### Subjects

The family was identified in the process of a multisite linkage consortium to collect families with bipolar disorder. The ascertainment and diagnostic methods have been described elsewhere^9^. Written informed consent was obtained from all participants. The study was approved by the University of California, San Diego, Human Research Protection Program. A structured interview using the Structured Clinical Interview for DSM-III-R (SCID) was performed, and the consensus diagnoses were made by a panel of experienced clinicians by reviewing the results of SCID, medical records, and other available information from family informants whenever possible. Autosomal dominant tubulo-interstitial kidney disease (ADTKD), previously known as medullary cystic kidney disease, was diagnosed by the treating nephrologists as described^9^. Detailed phenotype information can be found in Supplementary Fig. 1.

### Linkage analysis

Blood was drawn from 13 members, spanning three generations, including both affected and nonaffected members. DNA was prepared from lymphoblastoid cell lines by phenol/chloroform extraction. Genotyping for linkage analysis was performed as described in a previous paper^14^. Linkage analysis was performed in Merlin, using a nonparametric exponential model.

### Whole genome sequencing analysis

Genome sequencing was completed at TGen using Illumina Genome Analyzer I, Genome Analyzer II and HiSeq 2000 platforms. Exome sequencing of the proband was completed to expand the coverage in coding regions. Alignment, quality control, and variant calling was completed using the pipeline developed for the 1000 Genomes Project. Quality control was completed using the Genome Analysis Toolkit (GATK). FASTQ alignments were aligned with BWA to Build 36 using a paired-gapped alignment. Duplicate pairs were marked using Picard. SNPs were called using the GATK toolkit and postfiltering processing was completed to eliminate any false positives. Linkage-disequilibrium based calling was completed using Beagle or MACH. A detailed table for the filtering parameters can be found in the supplemental materials (Supplementary Fig. 2).

### Reprogramming of lymphoblast cell lines (LCL)

LCL were cultured in a standard PBLC media using RPMI 1640, 10% fetal bovine serum and 1% antibiotic/antimycotic. LCLs were cultured in T-25 culture flasks until confluent, then reprogrammed using the Epi5 Episomal iPSC Reprogramming kit (Invitrogen). Cells were transfected as described in the provided protocol, and plated into Matrigel-coated 6-well plates. Reprogrammed LCLs were cultured in TeSR-E7 reprogramming media (StemCell Tech) for 2 weeks, replacing half the media daily, then transitioned into mTeSR1 iPSC culture media (StemCell Tech). iPSC colonies are expanded by daily complete media changes, then manually picked via pipette and replated into a new Matrigel coated plate.

iPSCs are expanded in mTeSR1 media and treated with ROCK inhibitor (Y-27632, StemRD) for the first day in culture. Media is then changed daily until cells reach 80-90% confluence. Cells are passaged using ReLeSR (StemCell Technologies) as a dissociation reagent. Cells are then frozen in mTeSR1 + 10% DMSO, or passaged and replated onto Matrigel.

### Neural induction and characterization

Neural induction is completed using the dual SMAD inhibition neural induction kit (StemCell Tech). High quality iPSCs are selected and passaged using ReLeSR and centrifuged to pellet. Cells are then resuspended into a single cell suspension in STEMdiff Neural Induction Medium (StemCell Tech) + 10 μM Y-27632. Cells are replated onto matrigel coated plates at a density of about approximately 1 × 10^6^ cells/mL in a 6-well plate. Media is replaced the following day, without the addition of Y-27632, and is replaced every day until cells are confluent, approximately 6–9 days later. The NSCs are then dissociated again using Accutase (StemCell Tech) as a cell dissociation reagent, and replated in STEMdiff Neural Induction Medium + 10 μM Y-27632. Cells are cultured as described above until at least passage 3. Cells can then be characterized, cryopreserved, or further expanded.

Immunocytochemistry staining for Nestin and Sox2 has been used to confirm NSC identity. These are well established markers for human NSCs. Evaluated cell lines are >90% Nestin positive, therefore are primarily NSCs and thus suitable for downstream analysis.

### Cell proliferation assay

Cells were plated in a 96-well plate at 10,000 cells/well, with three replicates per cell line per day. Proliferation was measured every day for 5 days using the MTT Cell Proliferation Assay (ThermoFisher) using the Quick Protocol Option. Cells were changed to phenol red free Neurobasal media immediately before being incubated and labeled with MTT for 2 h.

Formazan is produced upon reaction with live cells and is then solubilized using DMSO. Absorbance was measured using the Tecan Infinite 200 Pro.

### Neurite growth assay

Cells were plated at a density of 10,000 cells/well and cultured for two days. Each cell line was plated in 8 wells of a 24-well plate. On day 2 of culture, 2 wells/cell line were treated with 100 ng/mL nerve growth factors (NGF) and incubated overnight. Images were taken in two randomly selected spots per well. Using ImageJ32, the total number of cells per image were counted, followed by the number of cells with at least one neurite growth.

### qPCR

Neural Stem Cell RNA was extracted using the Zymo Quick-PCR Mini-Prep Kit. cDNA was generated using the Superscript III (ThermoFisher) protocol. qPCR was performed using the Taqman Gene Expression Assay for *NTRK1* on the CFX ConnectTM Real Time PCR system and analyzed using the accompanying software, Bio-Rad CFX Manager TM. *HPRT1* was used as the reference gene.

### Generation of the Ntrk1 conditional knock-in mice

To establish *NTRK1* E495K conditional KI mice, we employed a flip-excision (FLEx) switch to flip the mutant exon 12 and excise the wild-type exon 12 in conjunction with expression of Cre recombinase. To construct the targeting vector, homology arms were obtained from a BAC clone (RP23-452A12, BACPAC Resources Center) containing genomic DNA for mouse *NTRK1*. FLEX switch (#18925, Addgene) with loxP and lox2272 sequences was utilized. Mutant exon 12 was generated by site directed mutagenesis with PCR. Positive (pgk-neo-polyA) and negative (Diphteria toxin A fragment, DT-A) selections were carried out as described^15^. The targeting vector was linearized and electroporated into the mouse embryonic stem (ES) cell line CMTI-2 (C57BL/6-derived) with standard gene targeting procedure. Screening of the correctly targeted ES cell clones was performed with Southern blotting using P^32^-radiolabeled DNA fragments as a probe. Twenty-five out of 400 clones screened were correctly targeted ES cell clones. The ES cell clones were microinjected into mouse blastocysts (Balb/c-derived) to obtain chimeric mice. By crossing the chimeric mice with C57BL/6J mice, F1 heterozygous flox/+ mice were obtained. Germline transmission was confirmed by PCR using the tail DNAs. After obtaining flox/+ allele, the mice were mated with Nestin–Cre transgenic mice to introduce the FLEx recombination specifically into the brain, or mated with CAG–Cre transgenic mice^16^ to obtain the conventional KI mice. Ntrk1 conventional heterozygous knockout mice were produced via CRISPR/Cas9 technology^17^, and homozygotes were obtained by intercrossing of the heterozygotes. Nestin–Cre transgenic mouse line (B6.Cg^Tg(Nes-cre)1Kln/J^) was obtained from Jackson laboratory. All animal experiments were approved by the Wako Animal Experiment Committee, RIKEN, and were carried out in accordance with the approved guidelines and regulations. All other experimental procedures were approved by the RIKEN Wako Safety Center and were performed in accordance with the approved guidelines. All behavioral analyses were carried out with male mice (3-months-old).

### Tail suspension test

Thirty minutes before the tail suspension tests, mice were intraperitoneally injected with saline or physostigmine (0.2 mg/kg**;** Tokyo Chemical Industry, Tokyo, Japan). Tail suspension test was performed using an infrared ray sensor system (Taiyo Electric Co. Ltd., Osaka, Japan), which showed activity counts but did not provide immobility time^18^. We performed TST with another system by analyzing images captured with CCD camera (O’Hara & Co., Tokyo, Japan), which provided immobility time as well as activity counts^19^.

### ChAT staining and analysis

Mice were anesthetized, transcardially perfused and fixed with 4% paraformaldehyde (PFA). Whole brains were dissected and embedded in paraffine blocks. Five micrometer coronal sections were prepared in the basal forebrain (including Medial septum, Diagonal band of Broca, Nucleus basalis). Immunohistochemical (IHC) staining was performed using an anti-cholineacetyltransferase (ChAT) antibody (AB144P, Millipore). The number of ChAT positive cells were counted and the diameter of the major axis of the intense ChAT signals in the cell bodies were measured with Neurolucida software (MBF Bioscience).

### Western blotting

Mouse brain tissues (male, 3–6 months) were homogenized with a Potter-type homogenizer in ice-cold buffer (25 mM Tris-HCl, 150 mM NaCl, 1% NP-40, 1% Sodium deoxycholate, 0.1% Sodium dodecyl sulfate) containing protease inhibitors and phosphatase inhibitors. After centrifugation supernatants were recovered and quantified. Proteins were separated by SDS-PAGE, transferred to PVDF membranes, and subjected to western blotting with antibodies for NTRK1 (#06-574, Millipore), Choline acetyltransferase (ChAT; AB144P, Millipore), ERK and phosphorylated-ERK (pERK) (#9101 and #9102 respectively, CST), and beta-actin (A5441, Sigma-Aldrich).

As for the fluorescence western blot of pERK/ERK, 30 min after physostigmine or saline injection, mice (male and female, 3 months) were sacrificed with cervical translocation. Whole brain tissues were quickly removed, and hippocampi were dissected out from the brain on ice, frozen with liquid nitrogen, and kept at −80 °C until use. After thawing on ice, the hippocampi were homogenized in the buffer with a Potter-type homogenizer and centrifuged at 4 °C for 15 min. Supernatants were recovered, and protein amounts were quantified by BCA kit. Equal amounts of proteins were loaded onto an SDS-PAGE gel and subjected to western blotting with antibodies for pERK (#9102, CST) and ERK (#9101 and #9107, CST) antibodies, and for fluorescence western blot, Cy5 or Alexa488 conjugated antirabbit or antimouse IgG antibodies (CST), respectively. The images were obtained and analyzed by FX fluorescence imager (Bio-Rad).

### Analysis of the dendrite morphology

Primary hippocampal neurons were prepared from embryos (embryonic day 16.5) of each genotype^20^. The primary neurons were plated into the 4 well slide chamber with a density of 20,000 cells/well and cultured at 37 °C in 5% CO_2_ in the minimum essential medium (MEM, Invitrogen) supplemented with B27 (ThermoFisher). After grown for 2 weeks, cells were fixed with 4% paraformaldehyde, permeabilized with 0.1% Triton X-100 and stained with anti-MAP2 antibodies (ab5392, Abcam) to visualize the dendrite morphology. Image acquisition was carried out with a confocal microscope (Olympus, Tokyo, Japan). Sholl analysis was performed with Neurolucida software.

### Statistical analysis

Statistical power was calculated by G*Power 3.1.9.2. Statistical analysis for the cell proliferation and neurite growth assays were completed using SPSS.

## Result

### Bipolar disorder is linked to chromosome 1q22 near the ADTKD-MUC1 locus

**Fig. 1:**
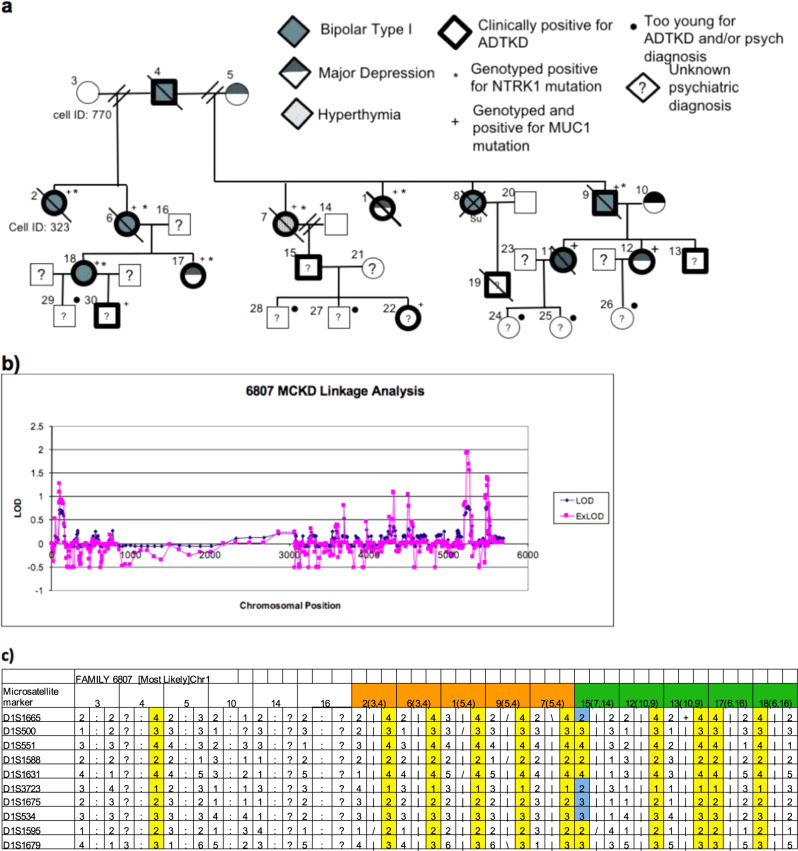
Identification of *NTRK1* mutation. **a** The pedigree of family 6807. **b** Linkage analysis plot for family 6807. **c** Microsatellite markers for chromosome 1, including *NTRK1* region. Haplotypes for. available members of the family (orange indicates 2nd generation, green indicates 3rd generation) and inferred haplotype for 1st generation father. Yellow columns indicate regions of homozygosity and blue regions indicate recombination events.

We previously described a large pedigree in which seven members had both ADTKD and mood disorder including five with bipolar disorder, one with recurrent major depression, and one with hyperthymia (Fig. 1a and Supplementary Fig. 1)^9^. Blood was drawn from the available family members and approximately 400 microsatellite markers were used to scan the genome. Subsequent linkage analysis identified suggestive peaks with lod scores between 1 and 2 on chromosomes 1, 12, 13, 19, and 22 (Fig. 1b). The linkage peak on 1q21 was of immediate interest as it maps to within several cM of the previously reported linkage region for ADTKD^10^. Stretches of homozygosity on each of these chromosomes was shared by all members of the second generation. When analyzing chromosome 1 markers, the majority of offspring of the third generation also received the same haplotype (Fig. 1c). Given the causative mutation of ADTKD was determined to be located on chromosome 1, the linkage analysis further suggested a possible co-segregation of mutations. Linkage analysis of chromosome 1 revealed that the SNP has a LOD score of 1.6, though not statistically significant or the largest in the genome scan, is noteworthy when considering the small sample size of a single family, the high degree of segregation with the two disease phenotypes and the proximity to a ADTKD gene.

### Whole genome sequencing identifies a likely functional variant in NTRK1

To search for the mutations in this locus, we performed whole genome sequencing in the proband (member 323) of this family. Exome sequencing was also performed to increase the coverage of coding regions. A total of 11,407 nonsynonymous coding sequence SNPs were identified. Three hundred and eighty-six of these SNPs were novel and not reported in the dbSNP or 1000 Genomes databases. Variants were filtered and mapped to within five linkage regions identified in the previously described linkage analysis. This resulted in 52 possible variants. Variants were then filtered for those genes expressed in either brain, kidney, or both. The resulting SNPs were then further filtered for those predicted to be damaging by SIFT or Polyphen2. SNPs identified in *NTRK1* were found to be the most promising candidates, considering its function in the cholinergic system.

A *NTRK1* mutation found in this family was a single nucleotide G to A variant (Chromosome 1: 156,875,639 [hg38]), causing substitution of glutamate to lysine at the 492 residue of TrkA protein (*NTRK1* E492K), and was predicted to be damaging by Polyphen2 with a score of 0.99. Sanger sequencing confirmed the mutation in all affected members of the family, where samples were available for testing. (Supplementary Fig. 3)

### E492K neural stem cells have reduced neurite growth

**Fig. 2:**
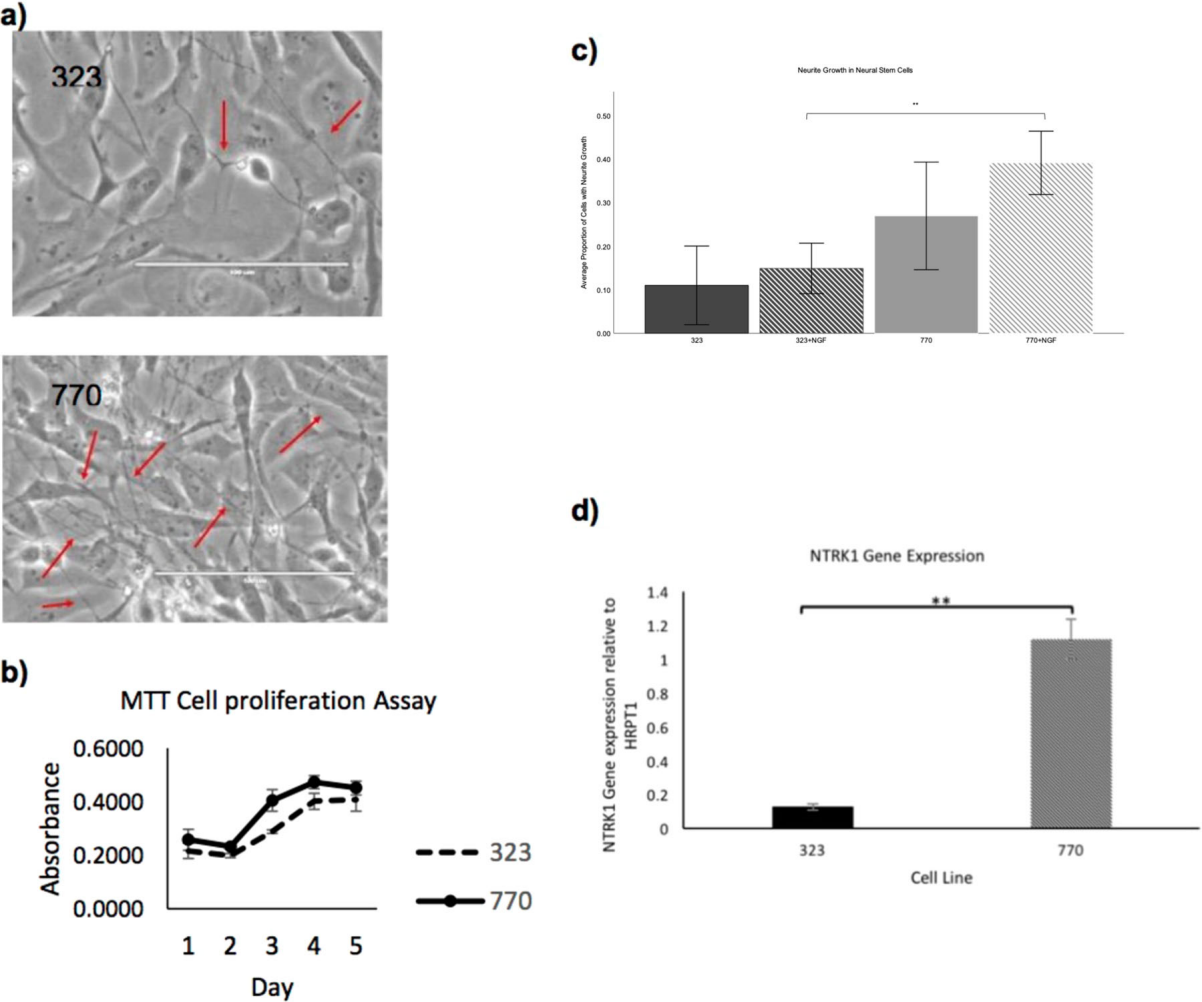
Functional analysis using neural stem cells. **a** NSC line morphology of E492K mutant line (323) and control line (770) with red arrows indicating neurite growth. NSC line 770 also appears to have denser growth of cells and more neurite outgrowth. **b** Cell proliferation measured by absorbance of formazan in NSC lines. (*n* = 3). **c** Proportion of neural stem cells in a given field with at least one neurite outgrowth with or without NGF treatment. Control cell line (770) has a significantly higher (One way ANOVA; *n* = 12, **p < 0.01) proportion of cells with neurite outgrowth when treated with NGF. **d** *NTRK1* expression of NSCs. *NTRK1* expression of the control cell line, 770, is significantly different (Independent *T*-test; **p < 0.01) when compared to the mutant cell line, 323.

We generated lymphoblastoid cell lines from one member of the pedigree with bipolar disorder and ADTKD as well as a healthy relative. Using the lymphoblastoid cells, we generated induced pluripotent cell lines (iPSCs). Neural induction of the iPSCs into neural stem cells (NSCs) was completed using dual SMAD inhibition. NSC line 323 is derived from the proband who carries the E492K mutation in TrkA. NSC line 770 is an unaffected relative and control. During the NSC expansion, the E492K NSC line, 323, appeared to grow more slowly than the control line 770 and seemed to produce less neurites (Fig. 2a).

In order to verify both of the observed phenotypes, NSCs were plated in equivalent densities in triplicate in 96-well or 24-well plates and a MTT cell proliferation assay was performed. NSC lines were plated in triplicate in 96-well plates and then measured for cell proliferation over the course of 5 days. There was a nonsignificant trend for reduced growth in the 323 line carrying the mutation (Fig. 2b).

NSC lines plated in 24-well plates were incubated overnight either with or without 100 ng/µL of nerve growth factor (NGF) in order to stimulate neurite growth. Each well was imaged twice, in randomly selected areas. The total number of cells were counted per image, as well as the total number of cells with at least one neurite growth. The E492K NSCs with NGF treatment had a significantly reduced proportion of cells with neurite growth when compared to the NGF-treated control cell line (Fig. 2c).

### E492K neural stem cells show a reduction in NTRK1 gene expression

In order to test the effect of the E492K mutation on *NTRK1* expression, RNA was extracted from each line for qPCR analysis. qPCR analysis revealed that the control cell line 770 appeared to express *NTRK1*, however the E492K NSC line appeared to have low or ablated *NTRK1* gene expression levels. The analysis was repeated, confirming the previous results, that *NTRK1* expression is reduced in the E492K mutant NSC lines (Fig. 2d).

### Establishment of brain-specific E495K knock-in mice

To further elucidate the role of the *NTRK1* E492K mutation, we generated a knock-in mouse line. The E492K mutation in human *NTRK1* corresponds to E495K in mouse *NTRK1*. Thus, we generated a E495K knock-in (KI) mouse to model the human disorder. TrkA is abundantly expressed with essential roles in peripheral tissues, which could affect behavioral analysis of the KI mouse^21,22^. Therefore, we introduced the point mutation specifically into the brain by using a Cre-mediated recombination to avoid systemic effects of the point mutation.

**Fig. 3:**
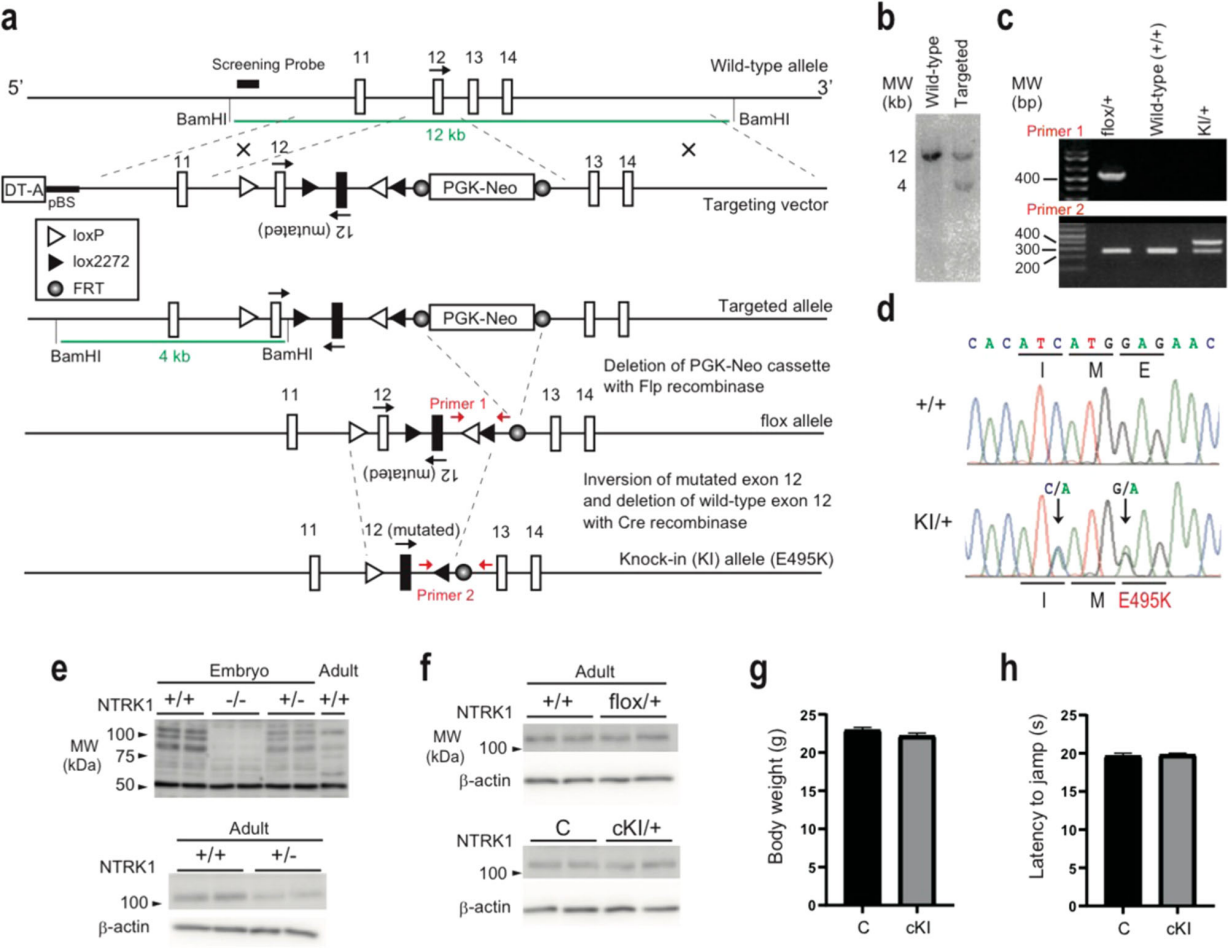
Generation of the brain-specific NTRK1 E495K knock-in (cKI) mice. **a** Gene targeting strategy. **b** Southern blot of the targeted ES cells. **c** PCR genotyping of the Ntrk1 recombined alleles. **d** Genomic DNA sequencing of the wild-type and KI alleles. **e** Validation of the anti-NTRK1 antibody specificity by western blots. Whole brain tissues were used for knockouts embryos (embryonic day 18). Cerebral cortices were used for the adult mouse samplesf) Expression levels of the NTRK1 protein among genotypes. C control. **g** Body weight. *N* = 13 for each genotype. **h** Hot plate test to assess the pain sensitivity. *N* = 13 for each genotype. Error bars, mean ± s.e.m.

To establish *NTRK1* E495K conditional KI mice, we employed a flip-excision (FLEx) switch to flip the mutant exon 12 and excise the wild-type exon 12 in conjunction with expression of Cre recombinase (Fig. 3a–d). The flox/+ mice were mated with Nestin-Cre (NesCre) transgenic mice to introduce the FLEx recombination specifically into the brain. The obtained flox/+; Nestin–Cre mice (Brain-specific heterozygous knock-in allele, cKI) were viable and appeared indistinguishable from control (+/+; Nestin–Cre) mice.

Expression levels of *NTRK1* protein in brain were checked by western blot (Fig. 3e, f). TrkA protein expression levels were similar among genotypes, that is E495K mutation did not affect the protein expression level itself. The antibody specificity was validated using the *NTRK1* knockout (embryo) and heterozygous mice (Fig. 3e). The TrkA protein expression levels in the brain appeared similar among flox/+, cKI, and control mice (Fig. 3f).

No significant difference was observed in their body weight at 3 months after birth (Fig. 3g; Control, 22.9 ± 0.36; cKI, 22.2 ± 0.35 g). Pain sensation was examined because *NTRK1* was known to be involved in nociception through peripheral nerves^21,22^. No significant difference was observed in a hot plate test under the common experimental condition (at plate temperature 54 °C, Fig. 3h; Control, 19.8 ± 0.18; cKI, 19.6 ± 0.35 s), which suggested that the cKI mice avoided the peripheral phenotypes observed in conventional *NTRK1* knockouts or heterozygotes such as insensitivity to pain.

### Physostigmine results in increased immobility in conditional KI

Consistent with the human data described above, it has been reported that acute treatment with physostigmine induces a depression-like phenotype in a tail suspension test (TST) in mice^23^. This supports the validity of the mouse to model cholinergic signaling in a depressive phenotype.

**Fig. 4:**
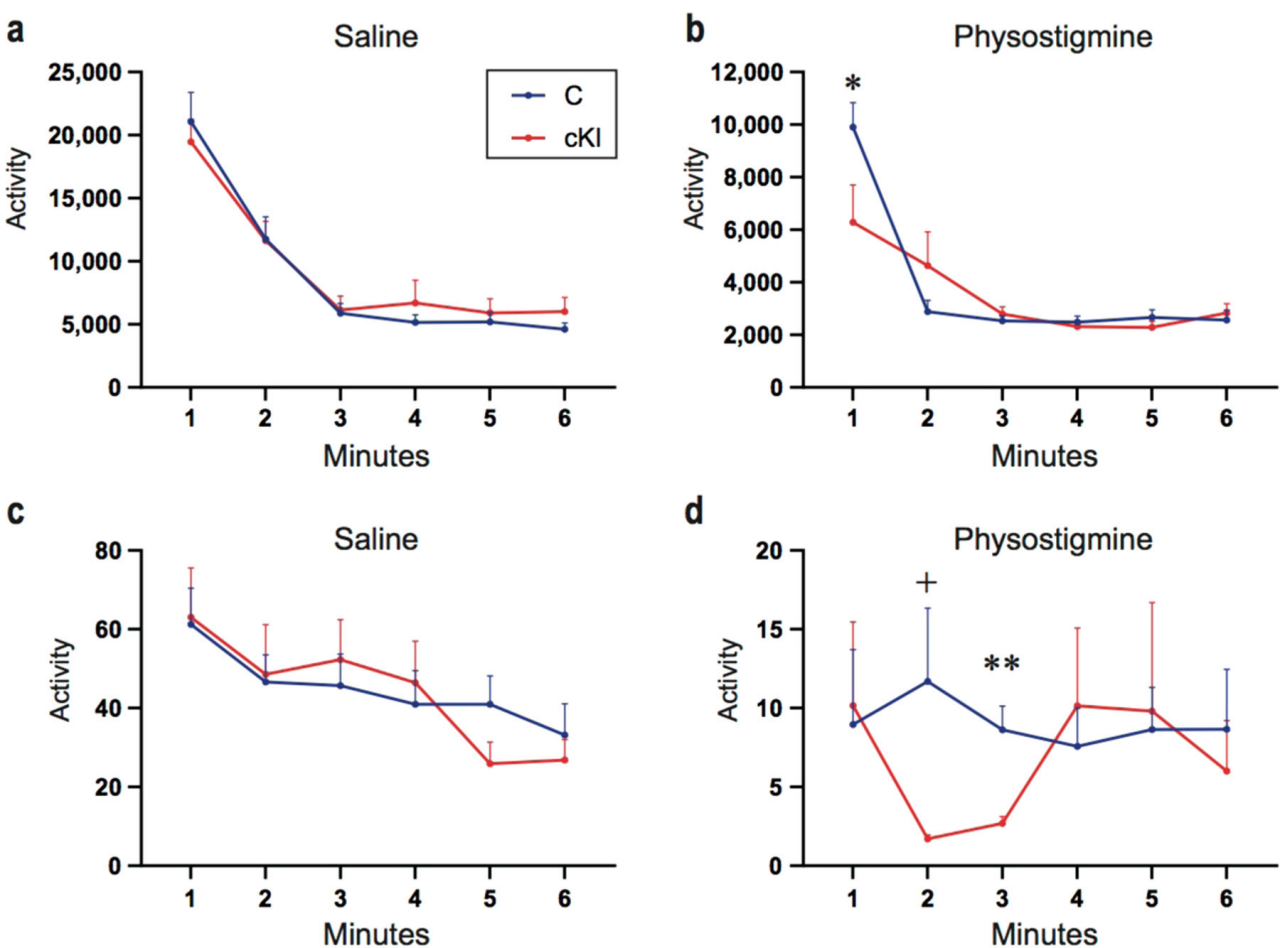
Depression like behavior under physostigmine administration in the Ntrk1 cKI mice. **a** Tail suspension test at the drug free state (saline). Control, *N* = 13; cKI, *N* = 13. **b** Tail suspension test after the physostigmine injection. Control, *N* = 13; cKI, *N* = 8. **c** Drug free state (saline) in an independent test. Control, *N* = 8; cKI, *N* = 8. **d** Increased depression-like behavior of the cKI mice with physostigmine was reproducibly observed in an independent test. Control, *N* = 8; cKI, *N* = 8. *p < 0.05, ^+^*p* = 0.05, **p < 0.005 (*t*-test, two-tailed); Error bars, mean ± s.e.m.

The TST was performed in order to investigate the role of the *NTRK1* E495K mutation in depression-like behavior mediated through cholinergic signaling. Without any treatment, no significant differences were observed between genotypes (Fig. 4a). However, physostigmine administration rapidly induced a significant decrease in activity in the TST in all genotypes. At 1 min, the cKI mice were significantly less active than the controls (*p* = 0.038; *t*-test, two-tailed) though after 3 min they were indistinguishable (Fig. 4b). These results were reproduced in an independent experiment (Fig. 4c, d). In these experiments the cKI animals were significantly less active than controls at the 2 and 3 min time points, though the total immobility time at 6 min did not show a statistically significant change (Fig. 4d).

In other behavioral tests including open field, elevated plus maze, and passive avoidance tests, no significant difference was observed.

### No difference in morphology of ACh neurons

**Fig. 5:**
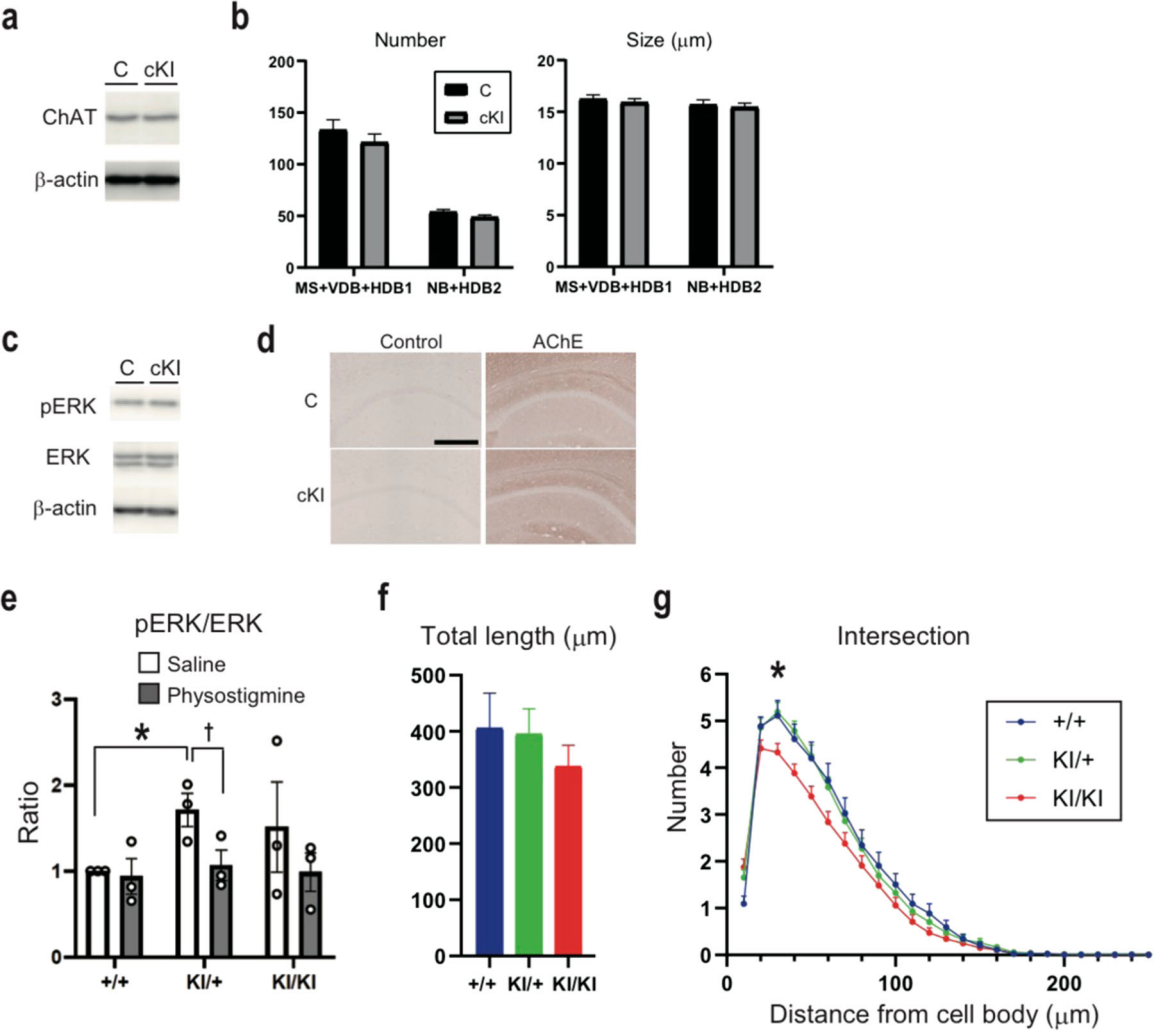
Development and maintenance of the basal forebrain cholinergic neurons were similar among genotypes; but postsynaptic defects were observed in the hippocampal neurons of the Ntrk1 KI mice. **a** Western blots of the ChAT protein using the basal forebrain tissue homogenates. *N* = 3 for each genotype, representative blots were shown. **b** Numbers and sizes of the basal forebrain cholinergic neurons, *N* = 3 pairs. MS medial septum, VDB ventral limb of the diagonal band of Broca; HDB horizontal limb of the diagonal band of Broca, NB nucleus of basalis. **c** Western blots of the pERK and ERK proteins using the basal forebrain tissue homogenates. *N* = 3 pairs, representative blots were shown. **d** Acetylcholine esterase immunohistochemistry in the hippocampal CA1 region. Bar, 250 μm. Acetylcholine esterase (AChE) immunohistochemistry in the hippocampal CA1 region. Anti-AChE antibodies (HPA019704, Sigma-Aldrich) were used. Bar, 250 μm. **e** pERK/ERK ratios of the hippocampus by fluorescence western blots using the tissue homogenates under saline or physostigmine administration. *N* = 3 pairs. *p < 0.05; ^†^*p* = 0.070 (*t*-test, two-tailed); Error bars, mean ± s.e.m. **f** Total length of the dendrites in the primary hippocampal neurons. **g** Number of intersections in the dendrites of the primary hippocampal neurons. **f**, **g** +/+, *N* = 44; KI/+, *N* = 84; KI/KI, *N* = 85 neurons were counted. *p < 0.05 (One-way ANOVA followed by Fisher’s PLSD post-hoc test); Error bars, mean ± s.e.m.

TrkA encoded by *NTRK1* is known to have important roles in the development and maintenance of the cholinergic nervous system^24^. We examined the expression levels of choline acetyltransferase (ChAT), a marker for cholinergic neurons, in basal forebrain by western blot. However, no significant differences were observed among genotypes (Fig. 5a). In addition, we checked the numbers and sizes of cholinergic neurons in basal forebrain by ChAT staining, but they were not significantly different among genotypes (Fig. 5b). Thus, the E495K mutation did not affect cholinergic neuron development, morphology or maintenance in the basal forebrain.

### Increased pERK/ERK in hippocampus

ERK is a MAP kinase and part of one of the downstream signaling cascades of *NTRK1*. However, expression levels of phosphorylated-ERK (pERK) in the basal forebrain were not significantly different amongst genotypes (Fig. 5c). As cholinergic neurons in basal forebrain innervate the hippocampus, we examined AChE (Fig. 5d) and pERK (Fig. 5e) levels in this cholinergic target. pERK tended to be increased in the cKI mice.

### Decreased dendritic branching in hippocampal neurons

To examine the morphological phenotype of the innervation target of cholinergic neurons, dendrite morphology was examined by using primary hippocampal neurons from embryonic brain (E16.5) (Fig. 5f, g). The dendrites were traced using Neurolucida 360, and the dendrite morphology was quantified by Sholl analysis. Whereas the total length of dendrites was not different between genotypes, the number of intersections in dendrites was significantly smaller in KI/KI than KI/+ or +/+, suggesting lower branching in KI/KI neurons (Fig. 5g; +/+, 5.11 ± 0.33; KI/KI, 4.32 ± 0.19 µm).

## Discussion

By performing whole genome sequencing analysis of a family in which bipolar disorder and ADTKD co-segregate, we identified the E492K mutation of *NTRK1* that is linked with *MUC1* and co-segregates with these two diseases. The iPSC-derived NSCs carrying the *NTRK1* E492K mutation showed reduced neurite growth and reduced *NTRK1* RNA. The conditional knock-in mice carrying the relevant mutation in the brain showed depression-like behavior in response to physostigmine, which enhances cholinergic signaling. Whereas there were no detectable morphological abnormalities of cholinergic neurons in the mutant mice, basal pERK levels were increased in hippocampus, suggesting that the E492K mutation might cause altered downstream signaling of TrkA in adult hippocampus.

This study has several limitations, the largest being sample size. This mutation (Chromosome 1: 156,875,639 [hg38]) is registered in the gnomAD database (1- 156845431-G-A; rs144901788) and found in 124 of 282262 alleles (0.043%). Assuming an odds ratio of 10, which is still less than the known major genetic risk factors of psychiatric disorders such as 22q11.2 deletion for schizophrenia (odds ratio = 67.7)^25^ and loss of function mutations of SETD1A (Odds ratio = 20.0 calculated from SCHEMA browser) and given the statistical power to detect the association is 0.79, it may be possible to determine association of the SNP in a reasonably sized case–control study. However, given the limitation of low frequency of this SNP, family studies, such as the one presented here, are a sufficient method to study the mutation in the context of mood disorders. In this particular situation, perfect co-segregation of this mutation with ADTKD comorbid with mood disorders in this family provides a reasonable support for the role of this mutation in mood disorders in this family.

We did examine case–control data from a previous study (Ament et al.^4^), of 3014 cases and 1717 controls, however rs144901788 was not identified in any sample. However, we did observe this mutation in one case and one control, in another smaller data set of 992 cases and 480 controls. Though the statistical power to detect the association in this sample size with the same assumption is too small to draw a conclusion (0.47), the presence of this mutation in one affected individual, unrelated to the pedigree presented within this manuscript, might provide further evidence for this rare variant to play a possible role in bipolar disorder. While the presence of the SNP in a control sample appears to be contradictory, some research suggest a polygenic nature of BP^26^. Therefore it is possible that the SNP may work in concert with other variants to produce the bipolar symptoms, of which the identified case may not also possess. The family presented in this manuscript has numerous other abnormalities in their genome, which may amplify the effect of this SNP. Alternatively, the case presented here may have variants which confer protection, as protective genotypes have been identified in other studies^27,28^. Our lab is pursuing an investigation of these other identified case and control samples for future studies of this SNP and bipolar disorder.

An additional concern is the comorbidity of such a severe form of ADTKD could present with another limitation. The family presented in this study appears to have a more severe form of ADTKD that results in renal failure at an unusually young age, suggesting that the kidney dysfunction could impact mental symptoms as well. However, age of onset of psychiatric symptoms consistently appear well before the age of onset of kidney symptoms, suggesting a comorbidity of diseases, as opposed to a causal effect of one disease from another. (Supplementary Fig. 1a, b) In fact, the almost complete penetrance for both the *MUC1* and the *NTRK1* mutation and the segregation of both diseases through all four generations, seem to be unusually high, further substantiating the hypothesis that these are causative mutations for the diseases.

As previously mentioned, this mutation is reported in the gnomAD database, however, psychiatric information is not available on these individuals, therefore we are unable to determine whether the individuals carrying this mutation are also affected by a mood phenotype. This mutation has also been reported in the literature as linked to a patient with congenital insensitivity to pain with anhydrosis (CIPA)^29^. While the primary symptom of CIPA is the insensitivity to pain, other symptoms may also include intellectual disability, absence of sweating and recurrent fevers, however there is a vast heterogeneity of clinical features exhibited in CIPA. Though none of the members within the family have reported insensitivity to pain symptoms, affected members are heterozygous for the mutation, whereas the reported CIPA patient was homozygous. Moreover, many CIPA patients with *NTRK1* mutations have been reported to experience mood related symptoms, frequently described as hyperactive, irritable and emotionally instable^30,31^. This further suggests that *NTRK1* could play a substantial role in mood regulation.

*NTRK1* mRNA is expressed in cholinergic neurons in basal forebrain and also in non-cholinergic neurons in other regions such as paraventricular thalamic nucleus, which is also implicated in mood disorders^19,32^. TrkA encoded by *NTRK1* plays a role in survival and maturation of cholinergic neurons of the basal forebrain^33^. The cholinergic hypothesis of depression is supported by patients with depression that have a hypersensitivity to cholinergic muscarinic receptor agonists such as physostigmine^11,12^. Thus, we hypothesized that the *NTRK1* mutation may impair the development of cholinergic neurons. While there was a significant difference in neurite growth and gene expression in the human neural stem cells there was no detectable morphological abnormality of mature cholinergic neurons in the Ntrk1 mutant mice. However, the mice showed hypersensitivity to physostigmine which enhances cholinergic neurotransmission. The limited time (1–3 min) that physostigmine effected the observed behavior may be related to the short effect that physostigmine exerts in general. This suggests that the NTRK1 mutation altered the sensitivity of the neurons innervated by cholinergic neurons to acetylcholine. Although the precise mechanisms are not completely understood, upregulation of pERK in hippocampus, which is innervated by the basal forebrain cholinergic neurons, supports this possibility.

*NTRK1* is expressed not only in the brain but also in kidney and peripheral nerve. Mutations of *NTRK1* are known to cause congenital insensitivity to pain and this phenotype can be attributable to TrkA in peripheral nerve^34^. On the other hand, NGF-TrkA signaling may also play a role in kidney diseases^35^. Thus, it is possible that *NTRK1* mutation in this family might also contribute to the kidney disease in this family. Indeed, the phenotype of ADTKD in this family is far more severe than other families. For these reasons, we successfully generated a conditional knock-in mouse. The mice did not show the phenotype of altered pain sensitivity, either hyposensitivity or hypersensitivity, which supported the use of conditional knock-in mice.

It is puzzling whether the E492K mutation of *NTRK1* impairs TrkA function or causes hyperactivation of TrkA signaling. Initially, we assumed that the mutation may cause alteration of autophosphorylation and thereby alter postreceptor signaling, because the mutation site is close to the Y490, which is auto-phosphorylated upon binding with NGF. However, analysis of TrkA autophosphorylation sites did not reveal any differences between genotypes (data not shown).

The E492K mutation of *NTRK1* is assumed to impair TrkA function because homozygous E492K mutation can cause CIPA^29^. In the iPSC derived NSCs, *NTRK1* signaling seemed to be attenuated, with marked decrease of *NTRK1* expression. Review of the literature suggested that *NTRK1* expression may be regulated via a positive feedback loop, in which NGF and TrkA bind, leading to activation of the PI3K pathway, the binding of transcription factor Lhx8 to a TrkA enhancer region which then promotes the expression of *NTRK1*^36^. This study also identified the MAPK/ERK pathway as also involved in promoting *NTRK1* expression, however it is still unknown through which mechanism this occurs. The MAPK/ERK pathway can be activated via the upstream binding of SHC to TrkA, which occurs following the autophosphorylation of TrkA at Y490. However, this binding site may be impaired due to the close proximity of the E492K mutation to the SHC binding site, therefore potentially reducing downstream *NTRK1* expression. On the other hand, pERK was increased in the hippocampus of the mutant mice, where expression of *NTRK1* is lower in mutant neural stem cells. This could be due to maturation status of neurons, or caused by the difference between in vitro and in vivo. In the in vivo condition, compensatory mechanisms and neuronal environment might alter the downstream signaling.

More research must be completed to determine how a compensatory mechanism or positive feedback loop may be occurring. One potential pathway that may be affected is the BDNF-TrkB pathway, which activates MAPK/ERK signaling in developing hippocampal neurons, and has been frequently implicated in depression^37,38^. Additionally, NGF has been shown to be synthesized in hippocampal neurons^39^. Trk receptors and their respective neurotrophins share signaling mechanisms and complementary effects regarding the survival and maintenance of neurons, therefore it is reasonable to expect that the TrkA-NGF and TrkB-BDNF pathways may interact in novel processes. It may be that an initial deficiency in TrkA-NGF signaling in cholinergic neurons may result in a compensatory upregulated response in other pathways and regions, such as the hippocampus, creating an overall impaired cholinergic signaling system. This supports that the two findings in cellular and animal models demonstrate different aspects of consequence of the *NTRK1* mutation depending on the different developmental stages of the neurons.

Compared with BDNF-TrkB signaling, the role of NGF-TrkA signaling has not been extensively studied especially in the adult brain. Identification of the *NTRK1* mutation linked with bipolar disorder sheds light on the new role of NGF-TrkA signaling and its relevance to mood disorders. Considering that upregulation of BDNF-TrkB signaling is implicated in antidepressant treatment, further studies on the role of NGF-TrkA are warranted which may lead to a new treatment principle.

## Supplementary information

Supplemental Figures

## References

[1] Goodwin, F. K., Jamison, K. R. & Ghaemi, S. N. *Manic-Depressive Illness: Bipolar Disorders and Recurrent Depression*. (Oxford University Press, Oxford, 2007).

[2] Stahl, E. A. et al. Genome-wide association study identifies 30 Loci associated with bipolar disorder. *bioRxiv* 173062 10.1101/173062(2018). 10.1038/s41588-019-0397-8 PMC6956732 31043756

[3] Ikeda M, Saito T, Kondo K, Iwata N (2018). Genome-wide association studies of bipolar disorder: a systematic review of recent findings and their clinical implications. Psychiatry Clin. Neurosci..

[4] Ament SA (2015). Rare variants in neuronal excitability genes influence risk for bipolar disorder. Proc. Natl Acad. Sci..

[5] Kataoka M (2016). Exome sequencing for bipolar disorder points to roles of de novo loss-of-function and protein-altering mutations. Mol. Psychiatry.

[6] Craddock N (1994). Familial cosegregation of major affective disorder and Darier’s disease (Keratosis Follicularis). Br. J. Psychiatry.

[7] Nanko S, Yokoyama H, Hoshino Y, Kumashiro H, Mikuni M (1992). Organic mood syndrome in two siblings with Wolfram syndrome. Br. J. Psychiatry.

[8] Siciliano G (2003). Autosomal dominant external ophthalmoplegia and bipolar affective disorder associated with a mutation in the ANT1 gene. Neuromuscul. Disord..

[9] Kimmel RJ (2005). Cosegregation of bipolar disorder and autosomal-dominant medullary cystic kidney disease in a large family. Am. J. Psychiatry.

[10] Kirby A (2013). Mutations causing medullary cystic kidney disease type 1 lie in a large VNTR in MUC1 missed by massively parallel sequencing. Nat. Genet..

[11] Dulawa SC, Janowsky DS (2019). Cholinergic regulation of mood: from basic and clinical studies to emerging therapeutics. Mol. Psychiatry.

[12] Gillin JC (1991). The cholinergic rapid eye movement induction test with arecoline in depression. Arch. Gen. Psychiatry.

[13] Wohleb E, Gerhard D, Thomas A, Duman R (2016). Molecular and cellular mechanisms of rapid-acting antidepressants ketamine and scopolamine. Curr. Neuropharmacol..

[14] Greenwood TA (2012). Further evidence for linkage of bipolar disorder to chromosomes 6 and 17 in a new independent pedigree series. Bipolar Disord..

[15] Sassa T, Gomi H, Itohara S (2004). Postnatal expression of Cdkl2 in mouse brain revealed by LacZ inserted into the Cdkl2 locus. Cell Tissue Res..

[16] Sakai K, Miyazaki J (1997). A transgenic mouse line that retains Cre recombinase activity in mature oocytes irrespective of the cre transgene transmission. Biochem. Biophys. Res. Commun..

[17] Nakajima K (2016). Exome sequencing in the knockin mice generated using the CRISPR/Cas system. Sci. Rep..

[18] Yamanishi K (2015). Hepatocyte nuclear factor 4 Alpha is a key factor related to depression and physiological homeostasis in the mouse brain. PLoS ONE.

[19] Kasahara T (2016). Depression-like episodes in mice harboring mtDNA deletions in paraventricular thalamus. Mol. Psychiatry.

[20] Kaech S, Banker G (2006). Culturing hippocampal neurons. Nat. Protoc..

[21] Smeyne RJ (1994). Severe sensory and sympathetic neuropathies in mice carrying a disrupted Trk/NGF receptor gene. Nature.

[22] Silos-Santiago I (1995). Non-TrkA-expressing small DRG neurons are lost in TrkA deficient mice. J. Neurosci..

[23] Mineur YS (2013). Cholinergic signaling in the hippocampus regulates social stress resilience and anxiety- and depression-like behavior. Proc. Natl Acad. Sci..

[24] Scola G, Andreazza AC (2014). The role of neurotrophins in bipolar disorder. Prog. Neuropsychopharmacol. Biol. Psychiatry.

[25] Marshall CR (2017). Contribution of copy number variants to schizophrenia from a genome-wide study of 41,321 subjects. Nat. Genet..

[26] Mellerup E (2017). Combinations of genetic variants associated with bipolar disorder. PLoS ONE.

[27] Whalley HC (2009). A GRIK4 variant conferring protection against bipolar disorder modulates hippocampal function. Mol. Psychiatry.

[28] Debnath M (2013). The HLA-G low expressor genotype is associated with protection against bipolar disorder. Hum. Immunol..

[29] Davidson GL (2012). Frequency of mutations in the genes associated with hereditary sensory and autonomic neuropathy in a UK cohort. J. Neurol..

[30] Li N (2019). Heterogeneity of clinical features and mutation analysis of *NTRK1* in Han Chinese patients with congenital insensitivity to pain with anhidrosis. J. Pain. Res..

[31] Verhoeven K (2006). Recent advances in hereditary sensory and autonomic neuropathies. Curr. Opin. Neurol..

[32] Holtzman DM (1995). TrkA expression in the CNS: evidence for the existence of several novel NGF-responsive CNS neurons. J. Neurosci..

[33] Fagan AM, Garber M, Barbacid M, Silos-Santiago I, Holtzman DM (1997). A role for TrkA during maturation of striatal and basal forebrain cholinergic neurons in vivo. J. Neurosci..

[34] Indo Y (1996). Mutations in the TRKA/NGF receptor gene in patients with congenital insensitivity to pain with anhidrosis. Nat. Genet..

[35] Bonofiglio, R. et al. Nerve growth factor (NGF) and NGF-receptor expression in diseased human kidneys. *J. Nephrol*. **20**, 186–195 (2007). 17514623

[36] Tomioka T (2014). LIM homeobox 8 (Lhx8) is a key regulator of the cholinergic neuronal function via a tropomyosin receptor kinase A (TrkA)-mediated positive feedback loop. J. Biol. Chem..

[37] Kumamaru E (2008). Glucocorticoid prevents brain-derived neurotrophic factor-mediated maturation of synaptic function in developing hippocampal neurons through reduction in the activity of mitogen-activated protein kinase. Mol. Endocrinol..

[38] Castrén E, Rantamäki T (2010). The role of BDNF and its receptors in depression and antidepressant drug action: reactivation of developmental plasticity. Dev. Neurobiol..

[39] Acsády L, Pascual M, Rocamora N, Soriano E, Freund TF (2000). Nerve growth factor but not neurotrophin-3 is synthesized by hippocampal GABAergic neurons that project to the medial septum. Neuroscience.

