# Supplementary material for: Ntrk1 mutation co-segregating with bipolar disorder and inherited kidney disease in a multiplex family causes defects in neuronal growth and depression-like behavior in mice
Source: Transl Psychiatry. 2020 Nov 24;10:407. doi: 10.1038/s41398-020-01087-8 (PMC7687911; doi:10.1038/s41398-020-01087-8)
Supplement: Supplementary file 1 — Supplemental Figures [file 41398_2020_1087_MOESM1_ESM.pdf]

a)

Subject	Psychiatric Diagnosis	Comorbidities	Age of Onset (psychiatric symptoms)	Age of Onset (ESRD)
1	Major Depression	ADTKD; alcoholism/substance dependency; panic disorder	19	34
2	Bipolar Type I	ADTKD; alcoholism/substance dependency; psychosis; rapid cycling, anorexia; panic disorder	7	18
3	Generalized Anxiety Disorder	Alcoholism	n/a	none
4	Bipolar Type I	ADTKD	n/a	n/a
5	Major Depression	n/a	n/a	none
6	Bipolar Type I	ADTKD; alcoholism/substance dependency; psychosis; rapid cycling,	12	24
7	Hyperthymia	ADTKD; short sleep periods	n/a	35
8	Bipolar Type I	ADTKD; psychosis; alcoholism/substance dependency		
9	Bipolar Type I	ADTKD; alcoholism/substance dependency; psychosis	28	~30
10	Major Depression	n/a	n/a	none
11	Bipolar Type I	ADTKD; substance abuse	n/a	n/a
12	Major Depression	ADTKD	n/a	n/a
13	Unknown	n/a	n/a	n/a
14	Psychiatrically healthy	n/a	none	none
15	Psychiatrically healthy	ADTKD; Gout	n/a	n/a
16	Unknown	n/a	n/a	none
17	Major Depression	ADTKD; Anxiety; sleep and memory complaints	n/a	24
18	Bipolar Type II	ADTKD; Anxiety; Sleep and memory complaints	n/a	22
19	Unknown	ADTKD	n/a	n/a

b)

Age of ESRD			
**Family**	**N**	**Average**	**Stdev**
**6807**	13	33.46	9.51
**All other ADTKD**	239	43.52	13.77

Supplementary Figure 1. Clinical summary of patients. Autosomal dominant tubulo-interstitial kidney disease (ADTKD); End stage renal disease (ESRD); a) Summary of psychiatric clinical diagnoses for available family members and spouses of 1st- 3rd generation, including additional comorbidities, age of onset for psychiatric symptoms and age of onset for ADTKD symptoms. "n/a" indicates when information is unavailable and "none" indicates information is not relevant for the patient. b) Average age of ESRD in family 6807, is significantly lower when compared to all other known ADTKD cases. (p = 0.009933)

Filter Method	SNP Type	Genome	Exome
First Pass: all SNPs	Total	3,333,079	108,976
	TiTv Ratio (All)	2.05	2.21
	TiTV Ratio (novel)	1.73	1.61
	Ti Tv Ratio (dbSNP)	2.10	2.29
	No. in dbSNP 129 (%)	2,909,250 (87)	97,914 (90)
	Concordance with dbSNP (%)	99.87	99.79
	No. not in dbSNP	423,829	11,062
	No. not in 1000 Genomes	242,057	7,116
	No. not in 12 exomes	241,918	6,960
Second Pass: all Coding SNPs	All Coding Variants (CDS)	22,304	20,244
	TiTv Ratio (All)	2.82	2.86
	TiTV Ratio (novel)	1.80	1.72
	Ti Tv Ratio (dbSNP)	2.98	3.03
	Homozygote	8,910	8,232
	Heterozygote	13,330	12,008
	Synonymous	10,959	10,245
	Missense	11,281	9,995
	Nonsense	141	103
	Splice Site	310	267
Third pass: all Novel SNPs	Novel Coding Varinats (CDS)	2,087	1,767
	TiTv Ratio (novel)	1.87	1.52
	No. not in 1000 Genomes	1,001	1,000
	No. not in 12 exomes	932	860
	Synonymous	291	280
	Missense	639	580
	Missense in CCDS	393	431
	Homozygote or Compound Heterozygote	298	371
	Homozygote	8	12
	Heterozygote	289	359
	Nonsense	5	14
	Splice Site	6	8
	Linked to Disease (HGMD)	75	92
Fourth Pass: Linkage Region	Novel, Missense under linkage peaks	43	49
	Homozygote	0	0
	Heterozygote	43	49
	Linked to diesase (HGMD)	9	15
	Associated with Neurological condition	1	2
	Associated with Kidney Condidtion	2	3
	Complex Disease	4	7
	Mendelian Disease	3	3

Supplementary Figure 2. Sequencing and SNP filtering Summary A. Sequencing was performed over a number of platforms including Illumina Genome Analyzer I, Genome Analyzer II, and a HiSeq 2000. **B.** Multiple pass, hypothesis driven SNP filtering method was applied where all available databases was used to filter out common variants. We used dbSNP129, 1000 Genomes Project and common variants deposited to the public domain from 12 human exomes. Quality analysis indicated that the transition/transversion ratio of novel coding variants were under the 3.0 expected ratio, indicating challenges in calling variants correctly without false positive call inflation.

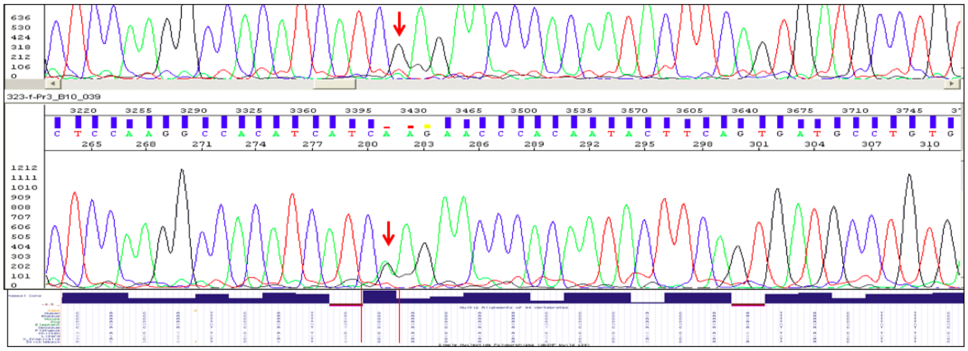

Supplementary Figure 3 Sanger sequencing confirms variant in affected family members
Family members and study participant were Sanger sequenced for the G>A nucleotide change and two traces are shown from data analysis. The trace on top indicates member #5 and associated homozygous to reference genotype (red arrow). Figure 1 shows the complete result of the Sanger sequencing validation. The locus is highly conserved across species visible under the Sanger traces, highlighted with red bars.
